# Pulmonary edema following diuretic therapy: A case report

**DOI:** 10.1097/MD.0000000000019180

**Published:** 2020-02-21

**Authors:** Lixia Liu, Qian Zhang, Tao Zhang, Xinhui Wu, Lixiao Sun, Bin Li, Xiaoting Wang, Yangong Chao, Zhenjie Hu

**Affiliations:** a Department of Intensive Care Unit (ICU), The Fourth Hospital of Hebei Medical University, Shijiazhuang, Hebei; b Department of Intensive Care Unit (ICU), Peking Union Medical College Hospital; c Department of Intensive Care Unit (ICU), The First Affiliated Hospital of Tsinghua University, Beijing, P.R. China.

**Keywords:** diuretic, pulmonary edema, ultrasound

## Abstract

**Introduction:**

Diuretics are a commonly used for the treatment of acute pulmonary edema. However, inappropriate administration of diuretic drugs can result in clinical treatment failure and cause acute pulmonary edema. This is due to rapid decreases in intravascular volume as a result of diuretic treatment. To date, the clinical phenomenon of inappropriate use of diuretics leading to acute pulmonary edema remains unexplored and unrecognized. Here, we report the first case of this problem—pulmonary edema following diuretic therapy.

**Patient concerns:**

A 71-year-old male patient who was intubated and transferred to the intensive care unit (ICU) due to respiratory failure was initially diagnosed with pneumonia as a complication of acute respiratory distress syndrome (ARDS). After treatments including antibiotics, lung protective ventilation strategies, and restrictive fluid management, his respiratory symptoms improved. However, the patient's dyspnea became more severe after experimental diuretic therapy.

**Diagnosis:**

A point-of-care ultrasound (POCUS) examination showed increased extravascular lung water retention during a hypovolemic state. After full examinations and analysis, the diagnosis of acute pulmonary edema was determined.

**Interventions:**

The most likely cause of acute pulmonary edema was left ventricular (LV) hyperdynamic status due to a hypovolemic status caused by excessive diuretic therapy. Consequently, we administrated intravenous fluids and a β-receptor blocker to the patient.

**Outcomes:**

Following these treatment, the patient's respiratory distress improved remarkably.

**Conclusion:**

We report the first case of pulmonary edema following diuretic therapy to stress the need of physicians to follow guidelines of clinical practice. Maintaining an appropriate volume status and treatment of β-receptor blockers is the key to reversing the progress of this adverse effect. In this process, POCUS is a reliable diagnostic tool to identify the cause of acute pulmonary edema and can increase the accuracy of clinical evaluations. It is likely that a wider use of POCUS will help physicians to obtain a faster, and more accurate, diagnosis of the etiology of acute pulmonary edema, thus allowing a more appropriate therapy.

## Abstract 2

Supplemental Digital Content is available in the text

## 1 Introduction

Acute pulmonary edema is a common complication in critically ill patients. The first 2 fundamentally different types of pulmonary edema which can occur in humans were reported as cardiogenic or non-cardiogenic pulmonary edema. Although they have distinct causes, it can be difficult to distinguish between the 2 due to their similar clinical manifestations, in particular when they develop simultaneously.^[1]^ Diuresis is the most common method used for treating pulmonary edema. However, inappropriate administration of diuretics can result in clinical treatment failure in acute pulmonary edema when we exactly don’t know its mechanism. We report a case of a special case of pulmonary edema following diuretic therapy. This is a result of rapid intravascular volume depletion secondary to inappropriate diuretic therapy. Pulmonary edema following diuretic therapy can be life-threatening depending on the time taken to diagnose. In this case, point-of-care ultrasound (POCUS) played a critical role in identifying the etiology of acute pulmonary edema.

## 2 Case presentation

**Figure 1:**
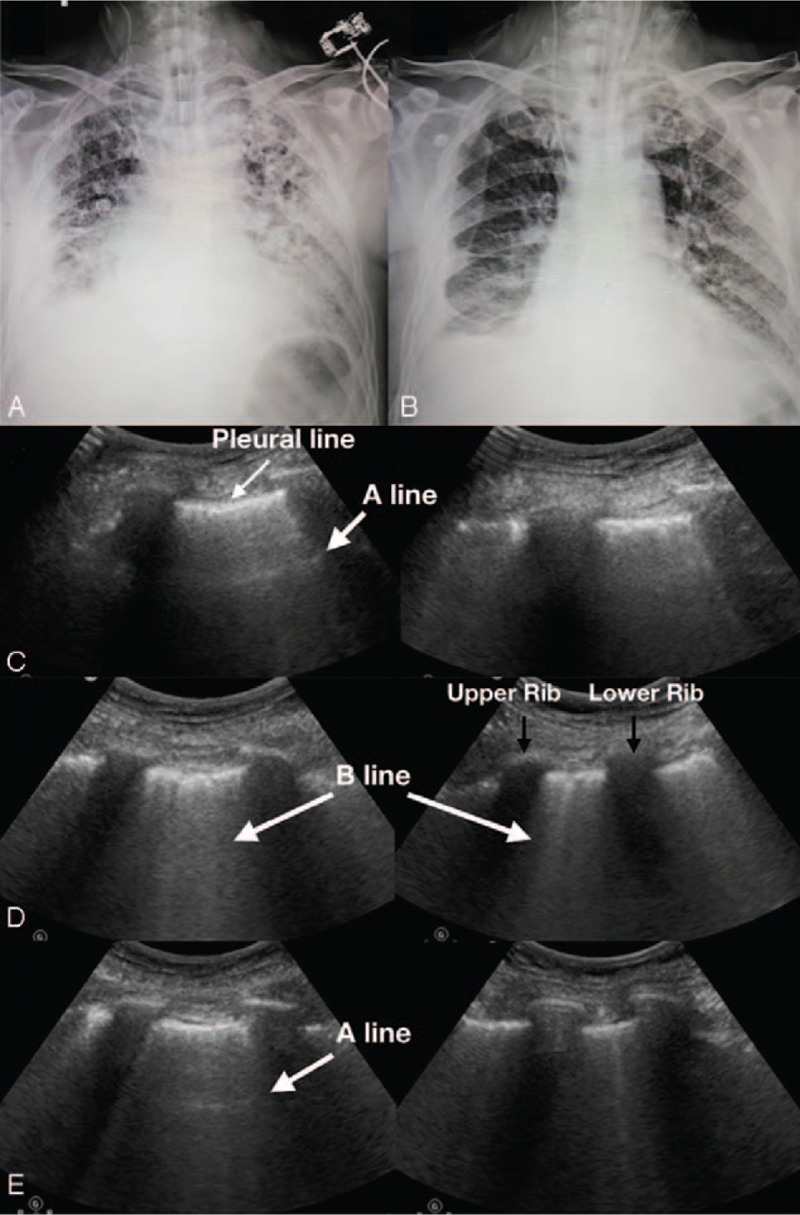
Imaging studies of chest x-ray and lung ultrasound. Chest x-ray showed bilateral diffuse and heterogeneous opacities on the first day of ICU admission (Panel A). Opacities diminished markedly within 1 week (Panel B) with bilateral A-line on the anterior chest (Panel C, left panel) and multiple B-line on the posterolateral chest wall (Panel C, right panel) evaluated by lung ultrasound examination. One day later, examination showed a marked increase of B lines bilaterally and a disappearance of A lines on the anterior chest (Panel D, left panel: upper point, right panel: lower point). After 24 hours of treatment, a repeated lung ultrasound revealed a marked decreased of B lines and A lines reappeared on the anterior chest. (Panel E, left panel: upper point, right panel: lower point). (The A line: the horizontal lines arising from the pleural line are separated by regular intervals that are equal to the distance between the skin and the pleural line. The A-line indicates air. The B line: multiple vertical comet-tail artifacts arising from the pleural line are spreading to the edge of the screen without fading and moving with lung sliding. It reflects the coexistence of elements with a major acoustic impedance gradient, such as fluid and air. Several B lines indicate increasing pulmonary fluid.). ICU = intensive care unit.

A 71-year-old man presented with subpleural tubercle on the right inferior lobe. Diagnosed as a non-small cell lung carcinoma, he underwent a lobectomy procedure. On the fifth postoperative day, the patient showed dyspnea. Upon examination, the patient had a heart rate of 122 beats/min, a respiratory rate of 30 breathes/min, and oxygen saturation of 86% to 90% on 10 L of oxygen via face mask. The patient was intubated and transferred to the ICU. He then received analgesia, sedation, and invasive mechanical ventilation. However, the patient remained in respiratory distress and his oxygen saturation sat at 84% while receiving 100% oxygen. A chest auscultation of the patient revealed rales bilaterally. A chest x-ray (CXR) showed bilateral diffuse and heterogeneous opacities (Fig. 1A). Once diagnosed with pneumonia causing the complication of acute respiratory distress syndrome (ARDS), the patient was treated with antibiotics, lung protective ventilation, and restrictive fluid management. His respiratory symptoms improved significantly within 1 week and the chest radiograph showed decreased pulmonary opacities (Fig. 1B). At this time, his cumulative fluid balance was negative 3050 mL.

**Figure 2:**
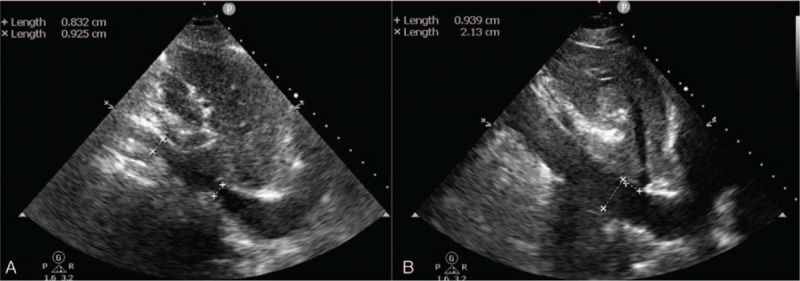
Inferior vena cava (IVC) diameter. IVC diameter was 0.92 cm with inspiratory collapse (Panel A). Video in Supplement 1. At post-treatment examination, IVC diameter increased to 2.1 cm (Panel B). Video in Supplement 2.

**Figure 3:**
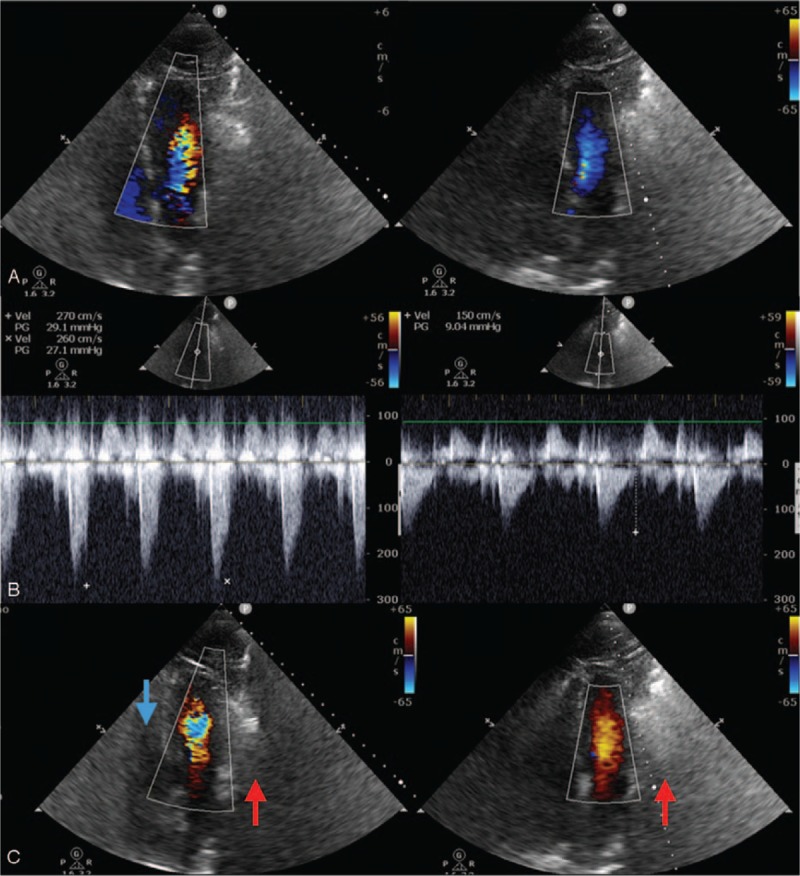
Echocardiographic imaging studies before (left panel) and after treatment (right panel). In a small hyperdynamic LV cavity, an aliasing of color flow doppler images was found across LVOT (panel A, left panel) with a maximal pressure gradient of 29 mmHg estimated by a continuous-wave doppler (panel B, left panel) in systole, and a paradoxical dynamic obstruction with a jet flow from the apex LV chamber to the basal was recognized in diastole (panel C, left panel) (video in Supplement 3). At post-treatment, the LV cavity enlarged with a laminar LVOT blood flow in systole (Panel A, right panel) and a laminar LV cavity blood flow from base to apex in diastole (Panel C, right panel) (video in Supplement 4). Meanwhile, the LVOT pressure gradient decreased to 9.0 mmHg (Panel B, right panel) in systole. LV = left ventricular, LVOT = left ventricular outflow tract.

After the patient experienced a state of great emotion and restlessness, he became extremely short of breath. Upon examination, his respiratory rate was 28 breathes/min, oxygen saturation had declined to 91% on mechanical ventilation with FiO_2_ at 50%, and chest auscultation demonstrated bilateral rales as previously. The diagnosis of acute pulmonary edema was determined. The patient was then immediately administered 40 mg of furosemide intravenously. In the ensuing 8 hours, fluid balance was measured at negative 970 mL however, the patient continued display dyspnea and worsening hypoxemia. His respiratory rate was then measured at 34 breathes/min, heart rate at 132 beats/min, and oxygen saturation was at 90% on mechanical ventilation with FiO_2_ of 70% and positive end-expiratory pressure (PEEP) of 7 cmH_2_O. POCUS showed an inferior vena cava (IVC) diameter of 0.92 cm with inspiratory collapse (Fig. 2A, video in supplementary file 1). The left ventricular cavity was small (LV end-diastolic dimension = 33 mm) with hyperdynamic left ventricular contraction (LV ejection fraction = 76%). These findings suggested a hypovolemic status and low cardiac preload. Most notably, in systole, an aliasing of color flow doppler images was seen across the left ventricular outflow tract (Fig. 3A left panel, video in supplementary file 3) and the maximal pressure gradient was 29 mmHg, estimated by a continuous-wave doppler flow (Fig. 3B left panel), suggesting an increased resistance of LV ejection. Meanwhile, a paradoxical dynamic obstruction with a jet flow from the apex LV chamber to the basal in diastole was observed (Fig. 3C left panel, video in supplementary file 3), with an early diastolic mitral inflow velocity of 1.1 m/s and an early diastolic mitral annulus velocity at the lateral side of 0.07 m/s (*E*/*e*′ratio = 15.7), implying that there was underlying LV diastolic dysfunction.^[2]^ Overall, this showed an elevated LV end-diastolic pressure and impaired LV filling. Accordingly, lung ultrasound evaluation showed increased B lines with a decrease of A lines in both lungs. All of this evidence demonstrated that extra vascular lung water increased despite a hypovolemic status after diuretic treatment (Fig. 1D).

Considered as the cause of acute pulmonary edema, the left ventricular diastolic dysfunction was due to a hypovolemic state accompanied with hyperdynamic left ventricular contraction. The patient received intravenous fluid therapy (Ringer lactate) for volume expansion, β-receptor blocker (intravenous esmolol) to mitigate increased sympathetic activity, minimize vigorous left ventricular contraction and improve left ventricular diastolic dysfunction and sedation (propofol) to eliminate stress responses and deep spontaneous breath. After 24 hours, the patient's fluid balance was positive 1390 mL. Meanwhile, the patient was stabilized again, respiratory rate was at 20 breathes/min, heart rate at 98 beats/min, and oxygen saturation at 98% on mechanical ventilation on FiO_2_ of 40% and PS of 12 cmH_2_O with PEEP of 5 cmH_2_O. Repeat POCUS evaluation showed the LV cavity had enlarged and the IVC diameter had increased to 2.1 cm (Fig. 2B, video in supplementary file 2), with a laminar left ventricular outflow tract (LVOT) blood flow in systole (Fig. 3A right panel) and a laminar LV cavity blood flow from base to apex in diastole (Fig. 3C right panel, video in supplementary file 4). The LVOT pressure gradient decreased to 9.0 mmHg in systole (Fig. 3B right panel) and the LV *E*/*e*′ ratio decreased to 9. These characteristics indicated that the hypovolemic status was corrected and left ventricular systolic and diastolic function had improved. A lung ultrasound examination showed decreased B lines compared with 24 hours beforehand and A lines reappeared in both lungs, implying extravascular lung water had decreased remarkably (Fig. 1E).

## 3 Discussion

Acute pulmonary edema is a common complication in critically ill patients. It is caused by increased hydrostatic pressure and increased permeability of vessels.^[1]^ In most cases, a restrictive fluid strategy in conjunction with diuretics remains the first line treatment of acute pulmonary edema. However, aggressive diuresis and rapid volume loss in the systemic circulation can lead to a series of complications.

In the present case, instead of reducing extravascular lung water then led to further deterioration of acute pulmonary edema. After comprehensive ultrasound examination, we considered that the cause lied in the mechanism that the heart and the lung only can work harder to increase oxygen delivery due to the hypovolemic status. Clinically, this manifests as hyperdynamic status of LV and deep and quick breaths.

The low cardiac preload due to the rapid volume loss resulted in hyperdynamic LV contraction. This leads to increased resistance of LV ejection during systole and difficulty with LV filling during diastole. The increased LV end-diastolic pressure and left atrial pressure were then transmitted to the pulmonary capillaries to increase pulmonary capillary hydrostatic pressure which pushed fluid out of the vessels and aggravated the hydrostatic pulmonary edema, which ultimately deteriorated the patient's pulmonary edema. Although the pulmonary arterial wedge pressure, estimated from *E*/*e*′, was not high enough to induce hydrostatic pulmonary edema, the effect of the hydrostatic pressure on the pulmonary edema may be amplified by high alveolar capillary permeability under certain conditions, such as pneumonia and ARDS. Fifty years ago, ARDS was first described as a form of respiratory failure in infants.^[3]^ This syndrome is characterized by an acute, diffuse, inflammatory lung injury, leading to increased alveolar capillary permeability. Eventually, the patient experienced exacerbated pulmonary edema and respiratory distress which triggered negative pressure pulmonary edema which attributed to the marked negative intrapleural pressure.^[4–6]^

Deep and quick breaths caused by pulmonary edema result in decreased thoracic pressure even into negative values, which will worsen hydrostatic pulmonary edema by increasing pulmonary capillary hydrostatic pressure. Firstly, the negative thoracic pressure increases right ventricular (RV) output by drawing more venous blood return to the RV^[4,6,7]^ and decreases LV output by elevating LV transmural pressure, contributing to the LV afterload increasing.^[8]^ Together, these 2 elements lead to increased pulmonary capillary hydrostatic pressure. Secondly, the negative thoracic pressure further increases trans-pulmonary capillary pressure. The combination of these elements in this patient resulted in increasing the severity of the hydrostatic pulmonary edema. Negative thoracic pressure also aggravates pulmonary permeability edema by increasing transpulmonary pressure which further exacerbates lung injury.

**Figure 4:**
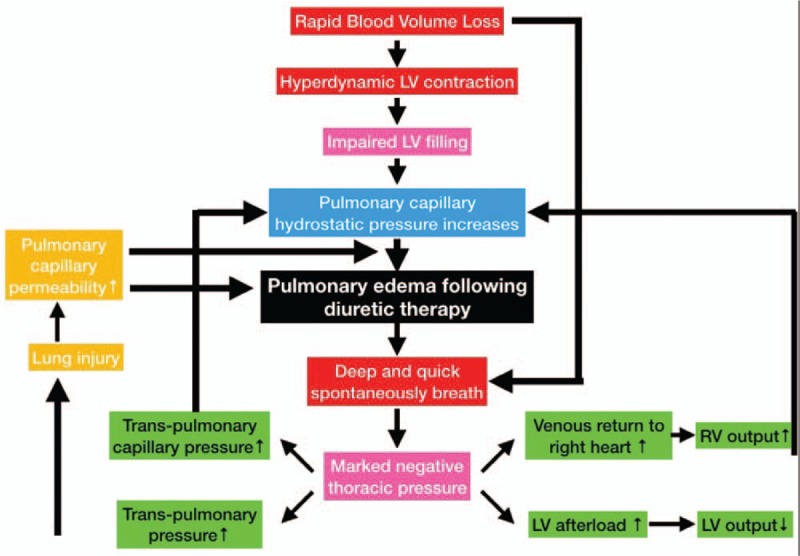
Mechanism of pulmonary edema following diuretic therapy.

Overall, pulmonary edema became more severe even in the volume depleted status. The patient improved remarkably after administration of fluids and β-receptor blockers. Although diuretics, vasodilators and inotropic agents are commonly used in clinical practice for the treatment of acute pulmonary edema, preliminary examinations with regard to pulmonary edema following diuretic therapy may guide us to better manage patients and avoid incorrect treatment. Hence, we first identify this type of acute pulmonary edema (Fig. 4). Maintaining an appropriate volume status and treatment with β-receptor blockers is the key to reversing the progress of pulmonary edema following diuretic therapy.

In addition, POCUS is an evolving tool in both emergency and critical care medicine. Using POCUS, clinicians can rapidly evaluate critically ill patients in real time rather than waiting for the availability of advanced imaging or invasive diagnostic procedures. In this context, a rapid bedside method to identify the cause of acute pulmonary edema could speed up the diagnosis, thus favoring early and appropriate therapeutic interventions. POCUS is effective in accurate diagnosis of pulmonary edema following diuretic therapy.

## 4 Conclusion

Pulmonary edema following diuretic therapy is a potentially life-threatening and under-recognized entity. A clear understanding of the mechanisms causing this may avoid incorrect treatment and help guide clinical practice. POCUS evaluation is invaluable for accurate identification and diagnosis of this type of pulmonary edema.

## Author contributions

**Investigation:** Xinhui Wu.

**Software:** Tao Zhang, Bin Li.

**Supervision:** Zhenjie Hu.

**Visualization:** Lixiao Sun.

**Writing – original draft:** Lixia Liu, Qian Zhang.

**Writing – review & editing:** Xiaoting Wang, Yangong Chao.

## Supplementary Material

Supplemental Digital Content

## Supplementary Material

Supplemental Digital Content

## Supplementary Material

Supplemental Digital Content

## Supplementary Material

Supplemental Digital Content
